# Inhibition of nicotinamide dinucleotide salvage pathway counters acquired and intrinsic poly(ADP-ribose) polymerase inhibitor resistance in high-grade serous ovarian cancer

**DOI:** 10.1038/s41598-023-30081-5

**Published:** 2023-02-27

**Authors:** Skye Alexandre Sauriol, Euridice Carmona, Molly L. Udaskin, Nikolina Radulovich, Kim Leclerc-Desaulniers, Robert Rottapel, Amit M. Oza, Stephanie Lheureux, Diane M. Provencher, Anne-Marie Mes-Masson

**Affiliations:** 1 Centre de Recherche du Centre hospitalier de l’Université de Montréal, Montreal, QC H2X 0A9 Canada; 2 Institut du Cancer de Montréal, Montreal, QC H2X 0A9 Canada; 3 Princess Margaret Cancer Centre, University Health Network, Toronto, ON M5G 1L7 Canada; 4 Department of Medical Biophysics, University of Toronto, Toronto, ON M5G 1L7 Canada; 5 Division of Medical Oncology and Hematology, University of Toronto, Toronto, ON M5G 2M9 Canada; 6 Division of Gynecologic Oncology, Université de Montréal, Montreal, QC H3C 3J7 Canada; 7 Department of Medicine, Université de Montréal, Montreal, QC H3T 1J4 Canada

**Keywords:** Cancer, Cell biology, Oncology

## Abstract

Epithelial ovarian cancer is the most lethal gynecological malignancy, owing notably to its high rate of therapy-resistant recurrence in spite of good initial response to chemotherapy. Although poly(ADP-ribose) polymerase inhibitors (PARPi) have shown promise for ovarian cancer treatment, extended therapy usually leads to acquired PARPi resistance. Here we explored a novel therapeutic option to counter this phenomenon, combining PARPi and inhibitors of nicotinamide phosphoribosyltransferase (NAMPT). Cell-based models of acquired PARPi resistance were created through an in vitro selection procedure. Using resistant cells, xenograft tumors were grown in immunodeficient mice, while organoid models were generated from primary patient tumor samples. Intrinsically PARPi-resistant cell lines were also selected for analysis. Our results show that treatment with NAMPT inhibitors effectively sensitized all in vitro models to PARPi. Adding nicotinamide mononucleotide, the resulting NAMPT metabolite, abrogated the therapy-induced cell growth inhibition, demonstrating the specificity of the synergy. Treatment with olaparib (PARPi) and daporinad (NAMPT inhibitor) depleted intracellular NAD+ , induced double-strand DNA breaks, and promoted apoptosis as monitored by caspase-3 cleavage. The two drugs were also synergistic in mouse xenograft models and clinically relevant patient-derived organoids. Therefore, in the context of PARPi resistance, NAMPT inhibition could offer a promising new option for ovarian cancer patients.

## Introduction

Epithelial ovarian cancer, especially the high-grade serous carcinoma (HGSOC) subtype, is the most fatal of all gynecological malignancies^1,2^, owing to its late detection, heterogeneous nature and resistance to treatment, particularly at recurrence^3–5^. Patients are usually treated with a combination of standard debulking surgery and chemotherapy^5,6^, and while initial response rates are often encouraging, relapse is observed in most cases^7,8^. Inhibitors of poly(ADP-ribose) polymerase (PARP) were first approved for ovarian cancer treatment in 2014^9^, and are now part of the standard of care for maintenance therapy in the first-line and recurrence settings for this disease^5,6,8,10^. While olaparib was first approved in the context of germline *BRCA* mutations for patients with recurrence, the emergence of new data led to PARP inhibitors (PARPi) being used earlier in patient care and, in the case of niraparib, independently from *BRCA* status^5,6,8^. However, despite their initial efficacy, acquisition of resistance to PARPi is observed in most cases, leading to subsequent relapse^11,12^.

PARPi mainly target PARP1, a highly expressed and ubiquitous protein responsible for synthesizing chains of poly(ADP-ribose) (PAR) directly onto its targets, including itself^13^. The PARP1 enzyme cleaves molecules of nicotinamide dinucleotide (NAD+) into nicotinamide (NAM) and ADP-ribose (ADPr), attaching the ADPr moieties onto its target^13^. PARP1 and its PARylation play a role in multiple essential pathways including DNA damage response and repair, chromatin remodeling and cell death^13^. PAR chains are subsequently broken down by poly(ADP-ribose) glycohydrolase (PARG) into NAM^14^. NAM is then recycled into NAD+ by the NAD+ salvage pathway via nicotinamide phosphoribosyltransferase (NAMPT) and nicotinamide mononucleotide adenylyltransferase (NMNAT)^15^. PARPi, such as olaparib, niraparib, rucaparib and talazoparib, mimic NAD+ and compete for the catalytic domain of PARP1, preventing synthesis of PAR chains and trapping it onto DNA^16,17^.

Multiple mechanisms have been proposed to explain PARPi resistance in HGSOC^11,12,18–20^. As PARPi are widely regarded to be most effective in homologous recombination (HR)-deficient tumors due to synthetic lethality, restoration of HR functionality is also the most commonly observed mechanisms of resistance in the clinic, usually through reversion or compensation via secondary mutations^11^. Alternatively, it has been reported that increased expression of efflux pumps such as ABCB1 reduces cellular levels of PARPi, limiting its effectiveness^18,21^. Mutations and loss of PARP1 have also been shown to induce PARPi resistance^22^; notably, mutations specifically in PARP1’s zinc finger domains cause resistance, as PARPi efficacy requires PARP1 to bind DNA^11^. Changes in dePARylation have been studied in the context of resistance to PARPi. Loss of PARG has been shown to counteract PARPi efficacy by allowing PAR accumulation and maintaining the function of PARP1^23^. Paradoxically molecular inhibition of PARG has also been demonstrated to effectively kill PARPi-resistant cells^14^.

Taken together, the evidence strongly suggests that PARPi resistance is multifactorial, and a full portrait of this complex phenomenon has yet to be drawn and therefore requires further investigation. We hypothesized that a more general approach to countering PARPi resistance might be attainable, taking into account the catalytic activity of PARP1 itself, where depleting its substrate NAD+ might render PARPi more effective against this enzyme. As the main pathway of NAD+ synthesis in cancer cells is the NAD+ salvage pathway^15^, we reasoned that the inhibition of this pathway’s rate-limiting enzyme NAMPT^24^ might circumvent PARPi resistance.

Here, we report that inhibition of the NAD+ salvage pathway abrogates acquired PARPi resistance in a variety of models that are otherwise highly diverse. The small molecule daporinad (FK866, APO866), a specific NAMPT inhibitor, is strongly synergistic with olaparib in all models tested, greatly potentiating the DNA damage- and cell death-inducing effects of olaparib. We demonstrate that synergy seems to be broadly applicable to PARP inhibitors and NAMPT inhibitors in general, and was also observed in in vivo xenograft and ex vivo organoid models. Since NAMPT inhibitors have already undergone clinical trials for other cancers, and based on the pre-clinical results presented here, these molecules could be reconsidered for HGSOC to effectively treat PARPi-resistant patients in a clinical setting.

## Results

### Cell line models were derived to study acquired PARPi resistance

**Figure 1:**
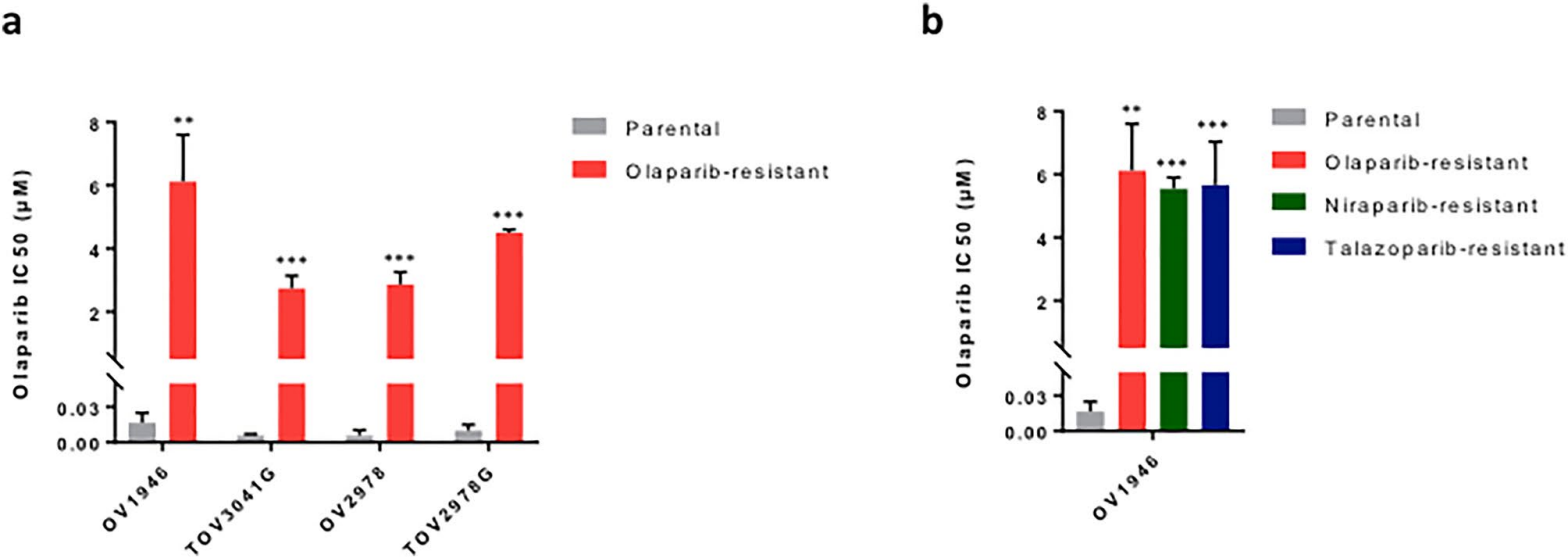
Olaparib sensitivity of the acquired resistance cell lines. Bar graphs comparing the olaparib IC_50_ values, evaluated by clonogenic assays, of each of the olaparib-resistant (-or) cell lines to that of their parental counterparts (**a**), and the olaparib IC_50_ values of the niraparib- and talazoparib-resistant (-nr, -tr) OV1946 cell lines compared to parental OV1946 (**b**). Experiments were repeated three times. Error bars represent SEM. Statistical significance was determined using Student’s *t* tests. *p < 0.05, **p < 0.01, ***p < 0.001.

In a previous study, our laboratory has published the olaparib sensitivity of a panel of HGSOC cell lines, ranging from very sensitive to strongly resistant^25^. To study acquired PARPi resistance, we selected six olaparib-sensitive cell lines (OV1946, TOV3041G, OV2978, TOV2978G, TOV1946 and OV2295)^25,26^ and generated resistant cell line models by exposing them to olaparib at increasing concentrations over an extended period of time (Fig. 1, Table 1, Supplementary Table S1). The developed cell lines gained the “olaparib-resistant” (or) suffix to denote their resistant phenotype and cell line of origin (for example: OV1946or). These cell lines showed high resistance to olaparib, with IC_50_ values (determined by clonogenic survival assay) in the range of 1.1 to 6.12 µM, 61- to 1154-fold higher than that of their parental counterpart (Fig. 1, Table 1, Supplementary Table S1). The IC_50_ values of our newly derived models were comparable to those of cell lines with intrinsic resistance or intermediate sensitivity to olaparib^25^. We then derived resistance to two other PARPi, niraparib and talazoparib, in our panel of sensitive cell lines using the same method and obtained resistant cell lines with a fold change ranging from 54.5 to 2288 (Fig. 1b, Supplementary Fig. S1a,b and Table 1, Supplementary Table S1). Interestingly, all tested cell lines showed cross-resistance to the different PARPi, in that our models are resistant to every PARPi tested, regardless of the inhibitor used to derive resistance (Fig. 1b, Supplementary Fig. S1a,b, Table 1, Supplementary Table S1). The resistant phenotype was stable, as evaluated by consistent IC_50_s after a freeze–thaw cycle^27^ and after at least five passages in absence of any PARPi. Cells were thus cultured in inhibitor-free medium after resistance was derived.

**Table 1:** Detailed olaparib IC_50_ values of acquired resistance cell lines.

Cell line	Parental IC_50_ (µM)	Resistant IC_50_ (µM)	Fold change
OV1946or	0.017 ± 0.008	6.12 ± 1.48	360.0
TOV3041Gor	0.006 ± 0.001	2.75 ± 0.40	458.3
OV2978or	0.006 ± 0.004	2.87 ± 0.39	478.3
TOV29878Gor	0.01 ± 0.005	4.01 ± 0.95	401.0
OV1946nr	0.017 ± 0.008	5.54 ± 0.35	325.9
OV1946tr		5.67 ± 1.36	333.5

Experiments were repeated three times.

Several mechanisms of PARPi resistance have been described in the literature^11,12,18–20^ and it is likely that our PARPi resistant cell lines have developed distinct resistance mechanisms, although a complete molecular characterization is warranted. They are therefore useful models to evaluate a more general approach to counter PARPi resistance. During cell line derivation, clonogenic assays were used to calculate IC_50_ values in order to evaluate drug sensitivity, as previously described^25^. However, this assay is not sufficiently high-throughput to perform drug combination array studies. Therefore, in subsequent experiments, live-imaging cell proliferation assays using 96-well plates (see Methods for details) were used instead.

### NAMPT inhibitors sensitize resistant cells to PARPi

**Figure 2:**
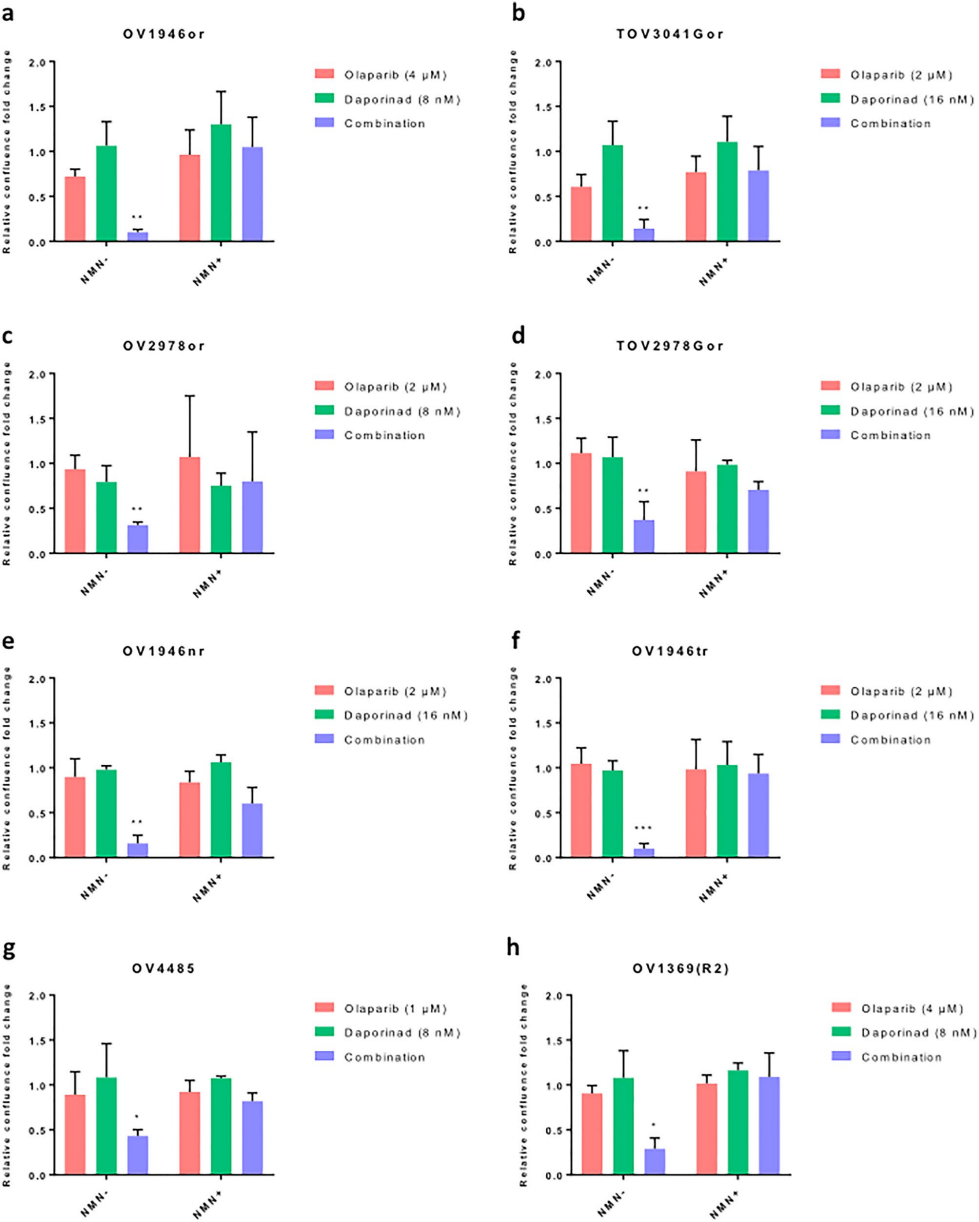
Combination of daporinad and olaparib in various resistant cell lines. Bar graphs comparing the relative confluence fold change at cell proliferation experiment end points after treatment with daporinad, olaparib or a combination of the two, in absence or presence of NMN. Experiments were repeated three times. Error bars represent SEM. Statistical significance was determined using Student’s *t* tests between the combination and each single agent, and only the highest p-value was illustrated. *p < 0.05, **p < 0.01, ***p < 0.001. Numerical values are detailed in Supplementary Figs. 2 and 6.

NAD+ is essential for PARP1 activity; it is used as a substrate to synthesize poly (ADP-ribose) chains, where PARP1 catalyzes the cleavage of this NAD+ into ADP-ribose and nicotinamide^28^. Nicotinamide is then recycled and used to synthesize NAD+ anew via the NAD+ salvage pathway, the main synthesis pathway for this coenzyme in cancer cells^15^. The rate-limiting enzyme of this pathway, NAMPT, has been targeted in pre-clinical studies and in clinical trials for treatment of multiple cancers, with mixed results^29,30^. Recent studies using NAMPT inhibitors as monotherapy in ovarian cancer cell lines have shown promising results to overcome drug resistance in certain contexts^31,32^. We evaluated the inhibition of NAMPT using the small molecule daporinad^33^ in four of our acquired resistance models (those with higher IC_50_ values to olaparib) and observed little to no effect. However, concurrently treating our cells with olaparib and daporinad significantly inhibited cell growth (Fig. 2a–f, Supplementary Fig. S2a–f, Supplementary Table S2) in all our acquired resistance cell lines, and the two drugs showed high synergistic potential at the tested concentrations (Supplementary Fig. S3a). We also show that in intrinsically PARPi-resistant HGSOC cell lines, OV4485 (*BRCA1*-mutated) and OV1369(R2) (Supplementary Fig. S1c–e), the combination of olaparib and daporinad is effective for inhibiting growth (Fig. 2g,h, Supplementary Fig. S2g,h, Supplementary Table S2), suggesting that inhibiting the NAD+ salvage pathway can prove effective to circumvent both acquired and intrinsic resistance. We further show that combining daporinad with niraparib or talazoparib also inhibits the growth of OV1946or (Supplementary Fig. S4), suggesting that daporinad could more broadly sensitize resistant cells to PARPi. Moreover, we show that combining olaparib with two other NAMPT inhibitors, OT-82 and KPT-9274, effectively reproduces the results obtained with daporinad, confirming that the observed synergy is a class effect, rather than a drug-specific effect (Supplementary Fig. S5a–d, Supplementary Table S3). Drug concentrations used were selected based on the strongest synergy combination observed for each cell line (see Methods for details) and are in the range used in previously published reports^25,31–35^. To confirm the specificity of the observed synergistic effects, we treated our cells with a combination of NAMPT inhibitors and olaparib, but with added nicotinamide mononucleotide (NMN), the resulting metabolite of the reaction mediated by NAMPT, in the cell culture medium. We show that adding NMN abrogates the effect of the combination on cell growth in both acquired and intrinsic resistance models (Fig. 2, Supplementary Figs. S3b, S5a,b,e,f, S6 and Supplementary Tables S3, S4), confirming that the synergy of this drug class combination is specifically due to NAMPT inhibition.

### The combination of olaparib and daporinad induces DNA damage and cell death

**Figure 3:**
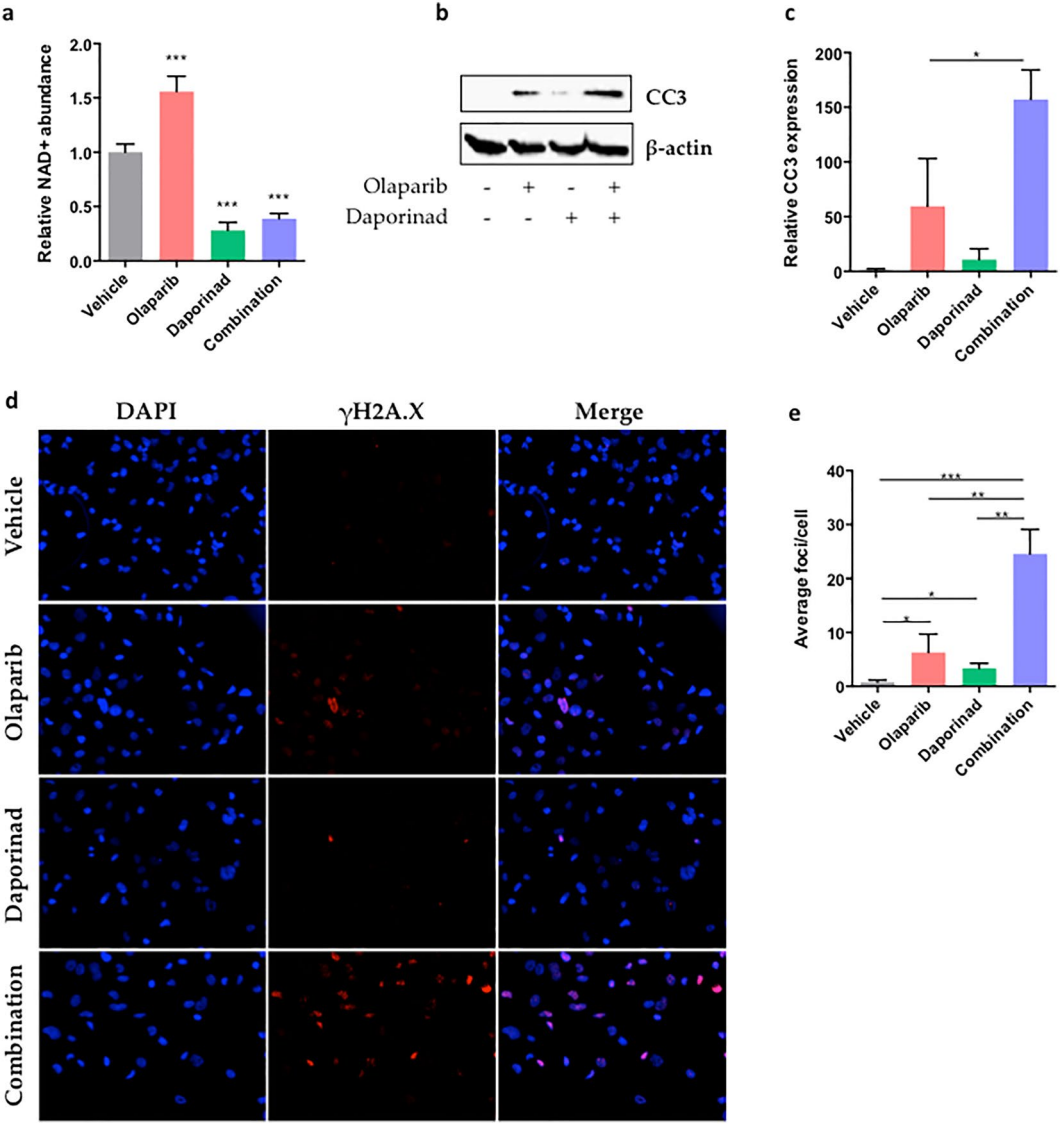
Effect of treatment on NAD+ metabolism and apoptosis. Relative intracellular NAD+  levels after 24 h of treatment (**a**). Western blot (**b**) and densitometry (**c**) of cleaved caspase-3 (CC3) expression after 5 days of treatment as a measure of apoptosis. Immunofluorescent staining (**d**) and quantification (**e**) of γH2A.X foci after 24 h of treatment as a measure of DNA damage. Experiments were repeated three times. Error bars represent SEM. Statistical significance was determined using Student’s *t* tests. *p < 0.05, **p < 0.01, ***p < 0.001.

It has been shown that inhibition of PARP1 with olaparib decreases NAD+ consumption^36^. This inhibition can be observed in our models, reflected by the observed increase in relative NAD+ levels in our olaparib-treated condition (Fig. 3a). On the other hand, the inhibition of NAMPT prevents cancer cells from regenerating NAD+ from nicotinamide, thus drastically reducing the intracellular concentration of this coenzyme^32^. We confirmed that this is the case by quantifying NAD+ in OV1946or cells after 24 h of treatment and show that the relative NAD+ levels in daporinad-treated cells are significantly lower than in untreated controls. The combination of olaparib and daporinad resulted in similar levels of intracellular NAD+ as treatment with daporinad alone (Fig. 3a). After five days of treatment, we show that olaparib alone induces cleavage of caspase-3 in OV1946or in spite of PARPi resistance, but that this effect is significantly increased with the addition of daporinad (Fig. 3b,c, Supplementary Fig. S7), indicating that this combination strongly induces apoptosis in resistant cells. As previously shown^37,38^, this increased level of apoptosis is likely to be at least partly due to an increase in DNA damage after treatment, supported here by the quantification of γH2A.X foci (Fig. 3d,e). Taken together, these data suggest that daporinad sensitizes cells to the DNA damaging effect of PARP inhibitors by depleting intracellular NAD+ , thus leading to increased cell death.

### Combining olaparib and daporinad slows tumor growth in vivo

**Figure 4:**
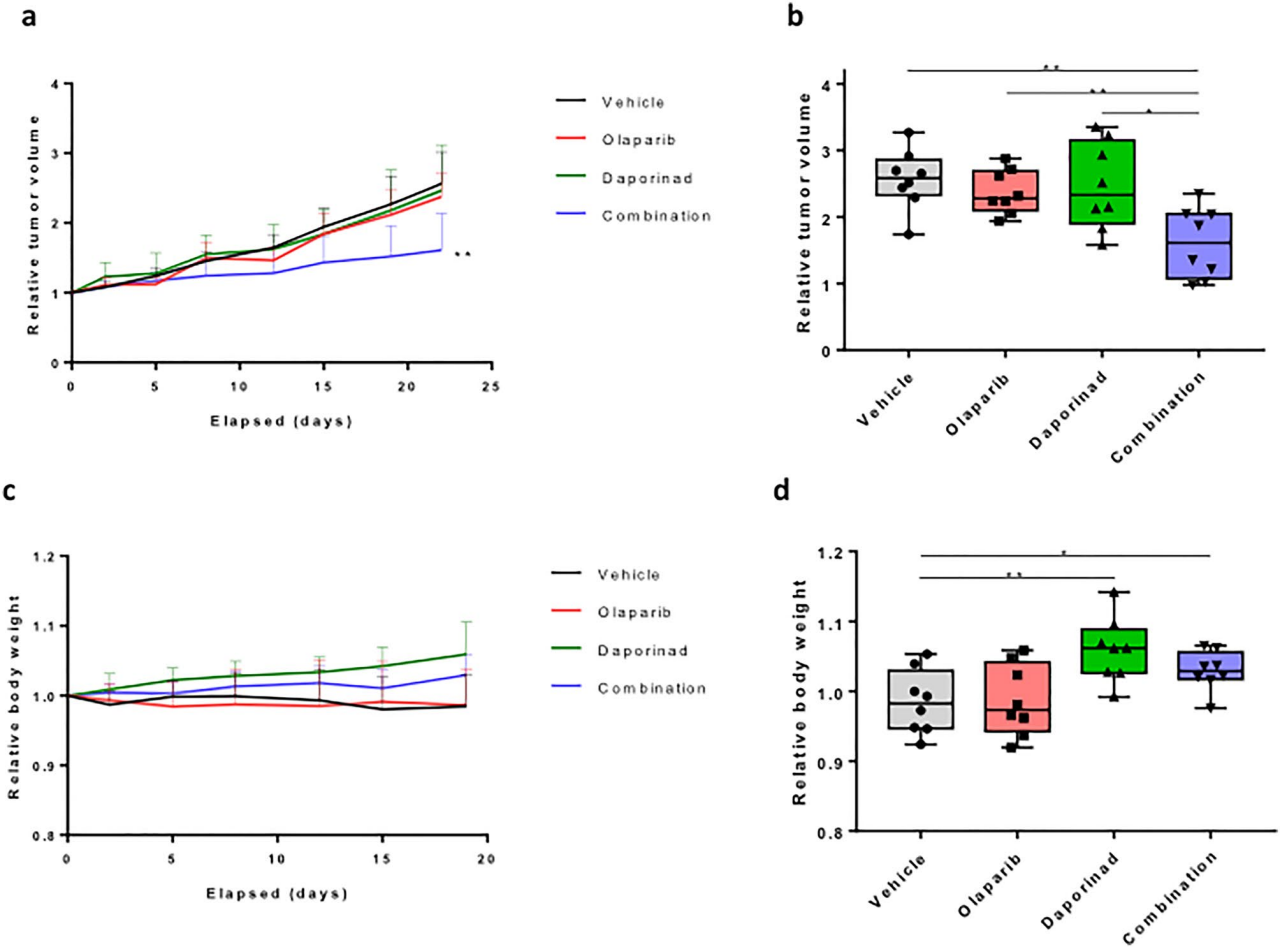
In vivo mouse xenograft models. Xenograft tumor growth over time per treatment condition (**a**), with box plots representing the relative tumor volume at end points (**b**). Evolution of mouse body weight over time (**c**) and at end points (**d**) were plotted. Each group consisted of 8 mice. Statistical significance was determined using Student’s *t *tests. *p < 0.05, **p < 0.01, ***p < 0.001.

To better evaluate the relevance of this combination for clinical treatment of resistant ovarian cancer, we used the OV1946 model for its capacity to form in vivo xenograft tumors^39^. We injected OV1946or cells in immunodeficient mice to form resistant subcutaneous xenograft tumors. When the tumors reached an average volume of approximately 400–500 mm^3^, the mice were treated with either olaparib, daporinad, the combination, or vehicle solution. We show that both drugs individually had no effect when compared to vehicle, but that the combination significantly slowed tumor growth (Fig. 4a,b, Supplementary Table S5). Furthermore, we show that the combination formulation is not toxic at the tested concentrations, as determined by mouse body weight variations (Fig. 4c,d), general monitoring of mouse health and macroscopic organ evaluation at time of sacrifice. Interestingly, the daporinad and combination conditions led to a significant increase in body weight after 15 days, compared to the olaparib and vehicle conditions. These data suggest that, in a clinical setting, combining olaparib and daporinad could prove effective at circumventing acquired olaparib resistance in HGSOC with minimal toxicity.

### Olaparib and daporinad are synergistic against primary patient organoid models

**Figure 5:**
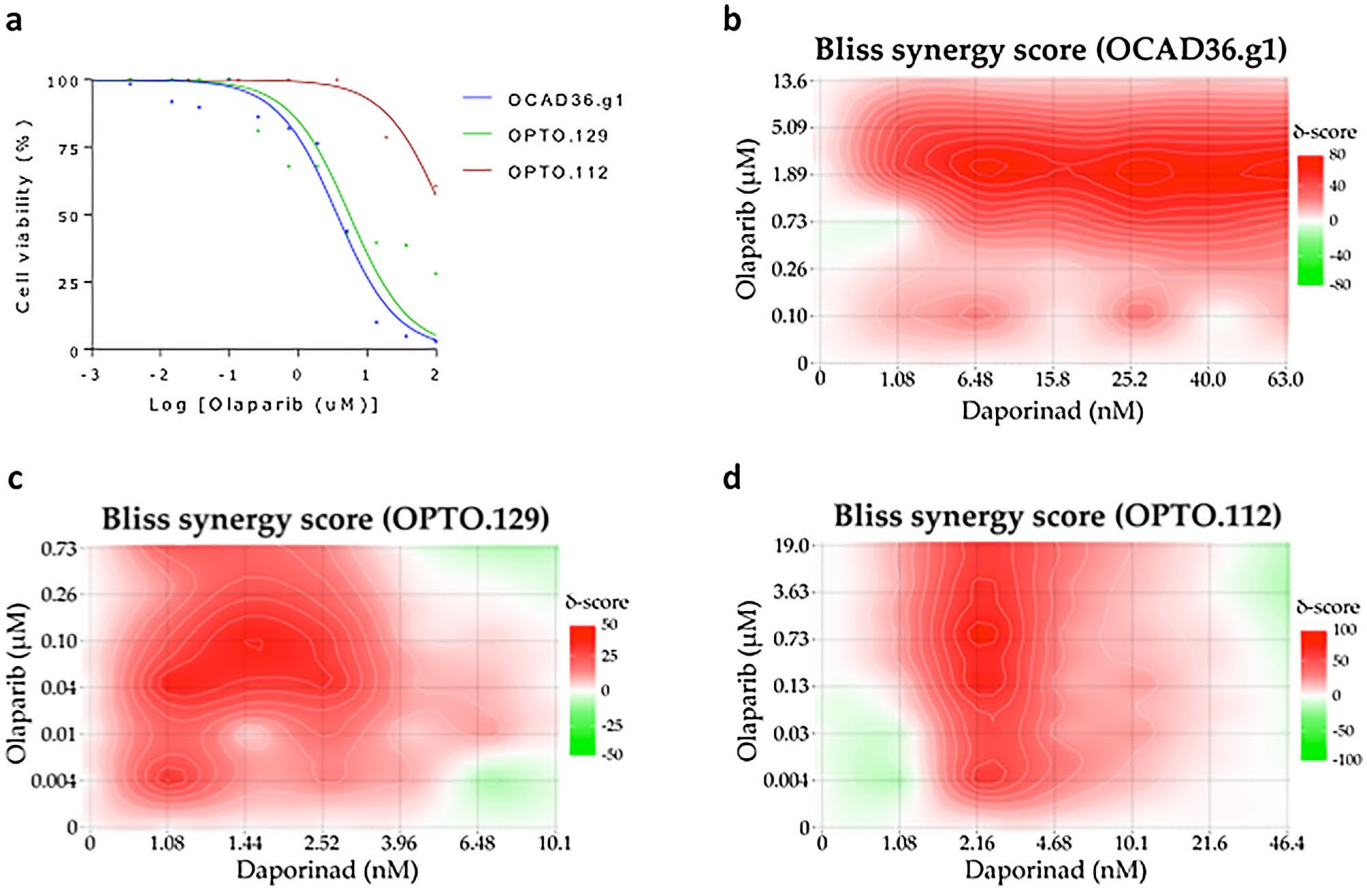
Primary patient organoid models. Olaparib sensitivity curves of the three organoid models (**a**). Bliss synergy maps of the combination for OCAD36.g1 (**b**), OPTO.129 (**c**) and OPTO.112 (**d**). Cell viability was measured by CellTiter-Glo 3D cell viability assay.

To further test the clinical relevance of the combination therapy, we evaluated the response of three primary PDO models (Fig. 5), the clinical characteristics of which are described (Table 2). Briefly, at the time of sampling, one patient (OCAD36.g1) was platinum-resistant, and another (OPTO.129) was platinum-sensitive. The third patient, from which OPTO.112 was derived, was also platinum-sensitive at sampling, but was treated with and relapsed on olaparib maintenance therapy (Table 2), indicating clinical olaparib resistance. When tested in vitro, two of the models (OCAD36.g1, OPTO.129) were sensitive to olaparib, and one (OPTO.112) was resistant (Fig. 5a). The combination of olaparib and daporinad proved synergistic and effective against all three of these models, including the model of clinical acquired PARPi resistance (Fig. 5b–d). These data further support the clinical relevance of this combination, including in a PARPi-resistant setting.

**Table 2:** Patient profiles for organoid models.

	OCAD36.g1	OPTO.129	OPTO.112
Stage	IIIc	IV	III
Subtype	High-grade serous	High-grade serous	High-grade serous
BRCA status	Wild-type	Wild-type	Wild-type
First-line treatment	NACT Carbo/Taxol Debulking surgery Carbo/Taxol	NACT Carbo/Taxol Debulking surgery Carbo/Taxol	Debulking surgery Carbo/Taxol
Second-line treatment	Gemcitabine (2 cycles) Caelyx (2 cycles)	Carbo/Taxol	Carbo/Taxol Olaparib maintenance
Third-line treatment	None	None	Debulking surgery Carbo/Taxol
Fourth-line treatment	None	None	Paclitaxel/Avastin
Time of sampling	After 1 cycle of Caelyx (second line treatment)	Initial debulking surgery	Second debulking surgery
Treatment received prior to sampling	Carbo/Taxol Gemcitabine Caelyx	Carbo/Taxol	Carbo/Taxol Olaparib
Sample type	Ascites	Tissue	Tissue

*NACT* neo-adjuvant chemotherapy, *carbo* carboplatin.

## Discussion

Our data show that HGSOC with acquired olaparib resistance can be rendered vulnerable to treatment by adding daporinad to the treatment regimen (Fig. 2a–f). These results are especially relevant in a clinical setting where olaparib is used for maintenance treatment, which often leads to the development of acquired PARPi resistance and subsequent relapse^40^. In this study, eight acquired resistance cell lines were used each with potentially distinct underlying mechanisms of acquired resistance. The diversity of models used supports a broader applicability for potentially different resistance contexts. Furthermore, we show that the combination proves effective in two intrinsically resistant cell lines, OV4485 and OV1369(R2), suggesting that the benefit of this treatment regimen might also be applicable in the context of first-line therapy, where certain patients initially fail to respond to treatment. This regimen is especially clinically relevant in the case of OV4485, a *BRCA1*-mutated cell line that displays resistance to PARP inhibition^25,41^, considering that, as previously mentioned, a portion of patients harboring a germline *BRCA* mutation do not respond to olaparib^42^. In total in this study we profiled cell lines derived from six different ovarian cancer patients which can be expected to have unique molecular profiles, and thus the results presented here suggest that a wide array of ovarian cancer patients, including *BRCA*-wild type patients, may benefit from this combination.

Our results for olaparib and daporinad seem to be more broadly applicable to PARP inhibitors and NAMPT inhibitors in general (Supplementary Figs. S4, S5, Supplementary Table S3). For PARPi, this includes niraparib and talazoparib, recently approved for use in breast cancer^43^ and currently undergoing clinical trials for ovarian cancer^44^; for NAMPT inhibitors, this includes OT-82 and KPT-9274^45^, both currently undergoing clinical trials, and especially interesting considering their oral route of administration, for clinical applications. Our data strongly suggest that the described combination is a class effect rather than a drug-specific effect and would remain relevant in the event of a shift towards newer and more effective PARPi or NAMPT inhibitors; further research would be warranted to confirm the benefit of the combination, especially in pre-clinical models.

Our experiments on primary PDO models demonstrate the clinical relevance of this combination therapy and is especially interesting in the case of the OPTO.112 model, which allows for the simulation of treatment of patients who have acquired PARPi resistance in a highly relevant clinical setting. The effectiveness of the combination on these organoid models, especially OPTO.112, offers strong evidence that this therapy could greatly benefit ovarian cancer patients.

Interestingly, it has been shown that daporinad may be effective in sensitizing triple-negative breast cancer (TNBC) to olaparib^38^. Indeed, Bajrami et al*.* have demonstrated that concomitant use of olaparib and daporinad has a greater effect on moderately olaparib-insensitive TNBC cell lines than olaparib alone. These results are pertinent in the context of intrinsic resistance, but the nature of the approval of olaparib as maintenance therapy highlights the importance of our findings on overcoming acquired PARPi resistance specifically, a current unmet need in clinical practice^11,12^. Taken together, the present work and the publication by Bajrami et al. suggest that a combination of olaparib and daporinad could be more broadly used to treat PARPi resistance in other malignancies for which these inhibitors are relevant, such as metastatic prostate cancer^46,47^, pancreatic cancer^48^ and small-cell lung carcinoma^49^. Further studies are warranted, especially considering the poor prognosis of some of these diseases^50–52^.

In addition to the synergistic potential of olaparib and NAD+ salvage inhibition, other aspects of NAD+ metabolism might be targetable to further potentiate the effect of PARP inhibition. We confirmed the specificity and involvement of the NAD+ metabolism in the combination of olaparib and daporinad by rescue experiments using NMN (the product of the activity of NAMPT), which was also observed when an NAMPT inhibitor was used as a single agent in other ovarian cancer cell lines^32^. In parallel, the inhibition of NAD+ and NMN import from the extracellular environment could be an interesting approach, as it is known that NAD+ can be found in the blood circulation^53^, and concentrations of NAD+ and its precursors, notably NMN, are dependent on diet and lifestyle^54,55^. It is established that extracellular NAD+ and its precursors can enter the cell; a study has shown that NAD+ uptake in mammalian cells is imported in a sodium-dependent fashion without being degraded extracellularly^56^. The same study has shown that extracellular NAD+ , NMN and nicotinic acid mononucleotide, but not NAM or nicotinic acid, could rescue daporinad-mediated mortality^56^, suggesting that NMN could also be imported into the cell. However, given that NAD+ and several of its precursors (such as NMN) cannot freely diffuse through membranes^57^, these would be actively imported, and the question of how this is accomplished is still being debated. Such a function for CD38 and CD73 had been proposed, where these secreted enzymes would convert extracellular NAD+ into importable precursor metabolites^58^, although recent research has shown that extracellular NAD+ could enhance PARP activity independently from CD73 and CD38^59^, suggesting that NAD+ can be imported through other means. Two recent studies have proposed a role for sodium-dependent channels SLC6A17 and SLC12A8 in the import of NAD+ and NMN respectively^60,61^. Following these data, inhibition of NAD+ and NMN import might further potentiate the synergy of the combination assay. However, as previously mentioned, blood concentrations of NAD+ and NMN in patients fluctuate with diet and lifestyle; these potential variations, in accordance with the possibility for NAD+ and NMN cellular import, might in part explain why daporinad showed low efficiency as a single agent in clinical trials. Further studies would be warranted to assess the effect of diet on the combination’s synergistic potential, and the value of adding inhibitors of NAD+ import in this context.

As for other pathways involving PARP1 and PARylation, inhibitors of PARG (enzyme that hydrolyzes PAR) have been exploited in cancer types with different sources of genomic instability, such as ovarian cancer. PARG inhibitors induce hyperPARylation of target proteins in DNA repair and the replication fork, increase DNA damage and exacerbate replication deficiencies [reviewed in^62–64^]. In a converse but similar way, PARPi abolish the PARylation of target proteins in these same processes, which will also induces DNA damage and aggravates replication deficiencies [reviewed in^62–64^]. Therefore, PARG inhibitors may complement PARPi action, and combination treatment has been shown to be synergistic in ovarian cancer cells^65^. More recently, the inhibition of Sirtuin1, a NAD+ -dependent deacetylase, has been shown to induce synthetic lethality in BRCA-mutated breast cancer cells^66^. This synthetic lethal interaction was associated with replication stress and increased cellular PARylation, in contrast to the decreased PARylation associated with PARPi synthetic lethality. In the present work, we opted to directly interfere with the catalytic activity of PARP by decreasing the pool of available NAD+ through the use of NAMPT inhibitors, thus augmenting the ability of PARPi to bind to PARP1, as PARPi compete with NAD+ (see model on Supplementary Fig. S8).

Our in vivo data demonstrates the efficiency of combining olaparib and daporinad in inhibiting the growth of a PARPi-resistant tumor (Fig. 4). Although our method does not perfectly simulate relapse following therapy resistance in vivo, our resistant cell line underwent a selection process in vitro following extensive treatment to olaparib, similar to what could have occurred in the context of olaparib maintenance therapy. Interestingly, the treatment of mice with daporinad alone or in combination caused a significant positive variation in the weight of mice (5.91% and 2.93% on average, respectively) (Fig. 4c,d). However, such a phenomenon was not observed with daporinad in previously published data using nude mice^38^; this suggests that the noted effect of daporinad on body weight might be due to the strain of mice used for the xenograft experiments.

Taken together, our data proposes an effective approach to address the problem of PARP inhibitor resistance. The heterogeneous nature of ovarian cancer and its propensity for resistance, both acquired and intrinsic, greatly contribute to its unmatched lethality amongst gynecological cancers^3,4^. We show that targeting the main biosynthesis pathway of PARP1’s substrate in cancer cells works to induce or restore PARPi sensitivity in all models tested, providing robust evidence of this combination’s usefulness both in vitro and in a preclinical model. The rising interest of NAMPT inhibitors and their ongoing clinical trials make them accessible for repurposing, and in spite of the mixed results of daporinad as a single agent, we believe that they have strong clinical potential for combination therapy, including for treatment of drug-resistant relapse in ovarian cancer patients.

## Methods

### Cell line culture conditions

HGSOC cell lines were cultured in 100 mm petri dishes (Sarstedt Inc., Nümbrecht, Germany) in OSE medium (WISENT Inc., St-Bruno, QC, Canada) supplemented with 10% fetal bovine serum (WISENT Inc.), 0.5 µg/mL amphotericin B (WISENT Inc.) and 50 µg/mL gentamycin sulfate (WISENT Inc.) (complete OSE medium). Plates were maintained at 37 °C in low oxygen conditions (7% O_2_ and 5% CO_2_). Cells were passaged at near confluence by trypsin 0.05% (WISENT Inc.) digestion. Cultures were discarded before the 20th passage, after which a fresh batch of cells was thawed for further experiments. For resistant cell lines, passages were counted from when stable resistance was confirmed.

### Acquired resistance cell line derivation

To derive our acquired resistance cell lines, the PARPi-sensitive HGSOC cell lines OV1946, TOV3041G, OV2978, TOV2978G, TOV1946 and OV2295^25,26,39,41,67^ (Cellosaurus Accession numbers CVCL_4375, CVCL_9T24, CVCL_A1SM, CVCL_9U73, CVCL_4062 and CVCL_9T13 respectively) were exposed to olaparib, niraparib or talazoparib 24 h after passaging at concentrations near their respective half maximal inhibitory concentrations (IC_50_). The cells were cultured in the presence of the inhibitor until their confluence lowered to 10%, at which time the culture medium was replaced with inhibitor-free fresh complete OSE medium. The cells were left to recover from treatment until reaching near-confluence, and subsequently passaged. Treatment was repeated at the same concentration post-passage until cell growth was not affected at the concentration used. Concentrations were then increased two-fold, and the process repeated until a sufficient level of resistance was reached. The stability of the resistance phenotype was determined by evaluating the IC_50_ of our resistant cell lines after at least five drug-free passages, as well as after a freeze–thaw cycle, as previously described^27^.

### Reagent and drug preparation

PARP inhibitors olaparib (MedChemExpress, Monmouth Junction, NJ, USA), niraparib (Abmole Bioscience Inc., Houston, TX, USA), and talazoparib (MedChemExpress), as well as NAMPT inhibitors daporinad (MedChemExpress), OT-82 (SelleckChem Chemicals, Houson, TX, USA) and KPT-9274 (SelleckChem Chemicals), were dissolved in dimethyl sulfoxide (DMSO) (MilliporeSigma, Burlington, MA, USA). β-nicotoninamide mononucleotide (NMN) (Sigma-Aldrich, St-Louis, MO, USA) was dissolved in sterile water.

### PARP inhibitor sensitivity assays

PARP inhibitor sensitivity was determined by clonogenic survival assays, as previously described^25,41^. 750 to 2000 cells were seeded per well in 6-well plates. The cells were treated with a range of PARP inhibitor concentrations 24 h after seeding, and the plates were incubated for 5 to 15 days, depending on cell growth, until colonies in the control (vehicle) wells were visible to the naked eye. The cells were then fixed for 10 min with cold methanol (Chaptec Inc., Montréal, QC, Canada), and then dyed for 10 min with a solution of 50% *v*/*v* methanol and 0.5% *m*/*v* methylene blue (Acros Organics, Thermo Fisher Scientific Inc., Fair Lawn, NJ, USA). Colonies were counted with a stereomicroscope, and the colony count for each concentration was represented as a mean percentage of the control wells. IC_50_s were calculated using the GraphPad Prism 7 software (GraphPad Software Inc., San Diego, CA, USA; www.graphpad.com). Each experiment was performed in duplicate and repeated at least three times.

### Drug combination assays

The effect of the combination between NAMPT inhibitors and PARP inhibitors was determined by live-cell imaging proliferation assays and confluence monitoring over time using the IncuCyte ZOOM System (Essen BioScience Inc., Ann Arbor, MI, USA). In a 96-well plate, 1000 to 2000 cells were seeded per well, and the cells were treated 24 h later with either the drug vehicle, a NAMPT inhibitor, a PARP inhibitor, or a combination of these two drugs, in presence or absence of NMN (0.5 mM). For each cell line, an array of concentrations were tested for each drug, and only the concentrations with the strongest synergy per cell line were retained for each drug for subsequent experiments. Selected concentrations for niraparib, talazoparib, OT-82 and KPT-9274 were 1 µM, 16 nM, 4 nM and 256 nM, respectively, for the OV1946or cell line. Concentrations for olaparib and daporinad ranged between 1 and 4 µM, and 8 and 16 nM respectively, depending on the cell line. Cell confluence was measured every four hours in each well for 5 to 12 days post-treatment, depending on the individual cell line growth rates, and each time point was represented as an average of the fold-changes from the initial confluence values per condition. Each experiment was performed in triplicate and repeated at least three times, except where otherwise indicated in figure legends. Curves were graphed using the GraphPad Prism 7 software. For OV1946or, synergy maps were generated using SynergyFinder^68^.

### Intracellular NAD+ quantification

OV1946or cells were treated with the drug vehicle (DMSO), 20 µM of olaparib, 20 nM of daporinad, or a combination of the two drugs in 100 mm petri dishes and incubated at 37 °C for 24 h. The treated cells were then harvested using trypsin, and 2 × 10^5^ cells were counted and pelleted per condition. Intracellular NAD+ levels were then quantified using the NAD+ /NADH quantification colorimetric kit (BioVision Inc., Milpitas, CA, USA) according to manufacturer instructions. Relative NAD+ levels were calculated as a ratio of absorbance compared to the vehicle condition. Experiments were performed in six replicates and repeated three times.

### Antibodies

For Western blots, cleaved caspase-3 (CC3) was detected using a primary anti-CC3 (Asp175) antibody (cat. #9664, Cell Signaling Technology, Whitby, ON, Canada) at a 1:1000 dilution, and a secondary peroxidase horseradish-conjugated goat anti-rabbit IgG antibody (MilliporeSigma) at a 1:2500 dilution. β-actin was detected using a primary anti-β-actin (AC-15) monoclonal antibody (cat. #ab6276, Abcam, Cambridge, UK) at a 1:10,000 dilution, and a secondary peroxidase horseradish-conjugated goat anti-mouse (H + L) IgG antibody (MilliporeSigma) at a 1:2500 dilution. For immunofluorescence, γH2A.X was detected using a primary anti-phospho-Histone H2A.X (Ser139) clone JBW301 antibody (cat. #05-636, MilliporeSigma) at a 1:1500 dilution, and a secondary Cyanine5-conjugated goat anti-rabbit IgG (H + L) cross-adsorbed antibody (Invitrogen, Waltham, MA, USA), at a 1:800 dilution.

### Western blot

OV1946or cells were treated with the drug vehicle (DMSO), 20 µM of olaparib, 20 nM of daporinad, or a combination of the two drugs in 100 mm petri dishes, and incubated at 37 °C. After five days of treatment, the cell supernatant was collected, and the treated cells were harvested with trypsin (0.05%). Adherent and suspended cells were pelleted together and rinsed, then lysed on ice for 30 min with lysis buffer (1% Triton X-100, 10% Glycerol, 50 mM Tris-Base pH 4.7, 2 mM EDTA, 150 mM NaCl) containing a protease and phosphatase inhibitor cocktail (Sigma-Aldrich, St. Louis, MO, USA). Total protein concentration was measured by Bradford protein assay (Bio-Rad Laboratories, Hercules, CA, USA) using a GENESYS 10S US-Vis spectrophotometer (Thermo Fisher Scientific, Waltham, MA, USA). 30 µg of total protein extract were loaded into precast Mini PROTEAN TGX™ 4–15% gradient Tris–glycine SDS–polyacrylamide 10-well gels (Bio-Rad Laboratories, Hercules, CA, USA), and migrated at 90 V for 65 min. The migrated proteins were transferred onto a Trans-Blot Turbo Midi 0.2 µm PVDF membrane (Bio-Rad Laboratories) with the Trans-Blot Turbo transfer system (Bio-Rad Laboratories) using the low molecular weight program. The membranes were blocked with a solution of 5% skim milk (Burlington, ON, Canada) and 0.05% Tween 80 (Sigma-Aldrich) in PBS (WISENT Inc.) for 60 min. The membranes were then incubated with primary antibody in the PBS-Tween-Milk solution overnight at 4 °C for CC3, or at room temperature for 60 min in the case of β-actin. The membranes were subsequently incubated with the secondary antibody for 60 min at room temperature, and the proteins were detected and imaged with Amersham™ ECL Prime Western Blotting detection reagents (GE Healthcare, Chicago, IL, USA) using the ChemiDoc MP Imaging System (Bio-Rad Laboratories). Densitometry was performed using the NIH ImageJ software^69^.

### Immunofluorescence

OV1946or cells were seeded on circular borosilicate glass coverslips (Fisher Scientific, Hampton, NH, USA) in 24-well plates, at a density of 40 000 cells per well. The cells were treated with the drug vehicle (DMSO), 20 µM of olaparib, 20 nM of daporinad, or a combination of the two drugs, and incubated at 37 °C for five days. The coverslips were then collected and the cells were fixed with 4% paraformaldehyde for 15 min, then permeabilized with 0.25% Triton X-100 (Sigma-Aldrich) for 20 min. The coverslips were subsequently blocked with a solution of 0.8% bovine serum albumen (BSA) (Sigma-Aldrich) and 4% donkey serum (Sigma-Aldrich) in PBS for 60 min, then incubated with the primary antibody overnight at 4 °C. The next day, the coverslips were incubated with the secondary antibody for 60 min at room temperature, then mounted onto microscope slides using ProLong Gold Antifade Mountant with DAPI (Invitrogen) and left to set overnight at room temperature, protected from light. Pictures were taken using a ZEISS Axio Observer Z1 microscope (Carl Zeiss AG, Oberkochen, Germany), and the quantification of foci per cell was performed using the NIH ImageJ software^69^.

### In vivo experiments

All animal studies (protocols C14008AMMs and C18010AMMs) were approved by the Institutional Animal Care Committee (IACC) of the CRCHUM (Montreal, Canada), following the conditions and guidelines established by the Canadian Council on Animal Care (CCAC) and in compliance with the ARRIVE guidelines. A suspension of 5 × 10^6^ cells in a solution of 50% Dulbecco’s phosphate buffer saline (DPBS) (WISENT Inc.) and 50% Matrigel basement membrane matrix (Corning Inc., Corning, NY, USA) was prepared for each mouse, for a total injection volume of 200 µL. The cell suspension was delivered into NOD.Cg-*Rag1*^*tm1Mom*^* IL2rg*^*tm1Wjl*^/SzJ (NOD rag gamma; NRG) mice (The Jackson Laboratory, Bar Harbor, ME) as a left gluteal subcutaneous injection, as previously described^41^. When the tumors reached an average volume of approximately 400–500 mm^3^, the mice were injected intraperitoneally with either 50 mg/kg of olaparib, 5 mg/kg of daporinad, a combination of these two drugs, or the drug vehicle, consisting of 10% DMSO (MilliporeSigma), 40% polyethylene glycol 400 (Sigma-Aldrich) and 50% DPBS (WISENT Inc.). The mice were housed under sterile conditions in a laminar flow environment with unrestricted access to food and water, and their weight and tumor xenograft volume was measured at least twice a week. Mice were treated once a day for 21 days, after which the animals were sacrificed in accordance with CCAC guidelines. At time of sacrifice, all organs were macroscopically evaluated by the animal technician together with the animal facility veterinary. Each group consisted of 8 mice.

### Organoids

This study used three human HGSOC patient-derived oragnoid (PDO) models (OCAD36.g1, OPTO.129 and OPTO.112). PDO models were generated, propagated and drug screened in the Princess Margaret Living Biobank core facility (https://www.livingbiobank.ca/). For drug screening, organoids were dissociated to single cells and seeded on top of a thin layer of Matrigel basement membrane matrix (Corning Inc.) in 384-well plate (2000 cells/well) 24 h prior to all drug treatments. For daporinad and olaparib combination drug assay, cells were treated with each drug alone and with combinations of various drug concentrations for 6 days. Daporinad and olaparib were applied at a range of 1 nM to 65 nM and 4 nM to 20 µM, respectively. Untreated control cells received vehicle alone. Cell viability was determined by CellTiter-Glo 3D Cell Viability Assay (Promega, Madison, WI, USA) according to the manufacturer’s protocol. Drug-response curves were graphed using the GraphPad Prism 7 software. Synergy maps were generated using SynergyFinder^68^.

### Ethics declaration and approval

The in vitro experiments using human-derived HGSOC cell lines were approved by the Comité d’éthique de la recherche (CER) of the Centre de recherche du Centre hospitalier de l’Université de Montréal (CRCHUM) (reference number 04.002). All animal studies (protocols C14008AMMs and C18010AMMs) were approved by the Institutional Animal Care Committee (IACC) of the CRCHUM (Montreal, Canada), following the conditions and guidelines established by the Canadian Council on Animal Care (CCAC) and in compliance with the ARRIVE guidelines. The human-derived organoid experiments were approved by the Ontario Cancer Research Ethics Board (OCREB) of the Ontario Institute for Cancer Research (OICR) (reference number 0875).

### Supplementary Information

Supplementary Information.

## Data Availability

All data generated or analysed during this study are included in this published article and its supplementary information files.

## Abbreviations

ADP: Adenosine diphosphate
ADPr: ADP-riboside
BRCA: Breast cancer DNA repair associated
BRCA1: Breast cancer 1 DNA repair associated
CC3: Cleaved caspase-3
CD38: ADP-ribosyl cyclase/cyclic ADP-ribose hydrolase 1
CD73: 5’-Nucleotidase
DMSO: Dimethyl sulfoxide
DPBS: Dulbecco’s phosphate buffer saline
DNA: Deoxyribonucleic acid
H2A.X: H2A histone family member X
HGSOC: High-grade serous ovarian cancer
HR: Homologous recombination
IC_50_: Half maximal inhibitory concentration
NACT: Neo-adjuvant chemotherapy
NAD+: Nicotinamide adenine dinucleotide
NAM: Nicotinamide
NAMPT: Nicotinamide phosphoribosyltransferase
NMN: Nicotinamide mononucleotide
NMNAT: Nicotinamide mononucleotide adenylyltransferase
NRG: NOD rag gamma
PAR: Poly(ADP-ribose)
PARG: Poly(ADP-ribose) glycohydrolase
PARP: Poly(ADP-ribose) polymerase
PARP1: Poly(ADP-ribose) polymerase 1
PARPi: PARP inhibitor
PDO: Patient-derived organoid
SLC12A8: Solute carrier family 12 member 8
SLC6A17: Sodium-dependent neutral amino acid transporter SLC6A17
TNBC: Triple-negative breast cancer
