# Author Correction: Inhibition of nicotinamide dinucleotide salvage pathway counters acquired and intrinsic poly(ADP-ribose) polymerase inhibitor resistance in high-grade serous ovarian cancer

**DOI:** 10.1038/s41598-024-60769-1

**Published:** 2024-05-01

**Authors:** Skye Alexandre Sauriol, Euridice Carmona, Molly L. Udaskin, Nikolina Radulovich, Kim Leclerc-Desaulniers, Robert Rottapel, Amit M. Oza, Stephanie Lheureux, Diane M. Provencher, Anne-Marie Mes-Masson

**Affiliations:** 1 Centre de Recherche du Centre hospitalier de l’Université de Montréal, Montreal, QC H2X 0A9 Canada; 2 Institut du Cancer de Montréal, Montreal, QC H2X 0A9 Canada; 3 Princess Margaret Cancer Centre, University Health Network, Toronto, ON M5G 1L7 Canada; 4 Department of Medical Biophysics, University of Toronto, Toronto, ON M5G 1L7 Canada; 5 Division of Medical Oncology and Hematology, University of Toronto, Toronto, ON M5G 2M9 Canada; 6 Division of Gynecologic Oncology, Université de Montréal, Montreal, QC H3C 3J7 Canada; 7 Department of Medicine, Université de Montréal, Montreal, QC H3T 1J4 Canada

Correction to: *Scientific Reports* 10.1038/s41598-023-30081-5, published online 27 February 2023

Since the publication of this work, Skye Alexandre Sauriol has changed their name from Alexandre Sauriol.

As a result, in the Author contribution statement the first sentence now reads:

“S.A.S. designed the in vitro and in vivo experiments, collected, analysed and interpreted the data, and drafted and revised the manuscript and figures.”

Additionally, Reference 26 has been corrected. It now reads:

Sauriol, S.A. *et al.* Modeling the diversity of epithelial ovarian cancer through ten novel well characterized cell lines covering multiple subtypes of the disease. *Cancers* **12**(8), 2222 (2020).

The original HTML and PDF versions of this Article, and its accompanying Supplementary Information file have been corrected.

