# Supplementary material for: Inhibition of nicotinamide dinucleotide salvage pathway counters acquired and intrinsic poly(ADP-ribose) polymerase inhibitor resistance in high-grade serous ovarian cancer
Source: Sci Rep. 2023 Feb 27;13:3334. doi: 10.1038/s41598-023-30081-5 (PMC9970983; doi:10.1038/s41598-023-30081-5)
Supplement: Supplementary file 1 — Supplementary Information. [file 41598_2023_30081_MOESM1_ESM.pdf]

Supplementary Information

Inhibition of nicotinamide dinucleotide salvage pathway counters acquired and intrinsic poly(ADP-ribose) polymerase inhibitor resistance in high-grade serous ovarian cancer

Skye A. Sauriol[1,2], Euridice Carmona[1,2], Molly L. Udaskin[3], Nikolina Radulovich[3], Kim Leclerc-Desaulniers[1,2], Robert Rottapel[3,4], Amit M. Oza[3,5], Stephanie Lheureux[3,5], Diane M. Provencher[1,2,6], Anne-Marie Mes-Masson[1,2,7]*

[1] Centre de recherche du Centre hospitalier de l'Université de Montréal, Montreal, QC H2X 0A9, Canada.

[2] Institut du cancer de Montréal, Montreal, QC H2X 0A9, Canada.

[3] Princess Margaret Cancer Centre, University Health Network, Toronto, ON, M5G 1L7, Canada.

[4] Department of Medical Biophysics, University of Toronto, Toronto, ON, M5G 1L7, Canada.

[5] Division of Medical Oncology and Hematology, University of Toronto, Toronto, ON, M5G 2M9, Canada.

[6] Division of Gynecologic Oncology, Université de Montréal, Montreal, QC H3C 3J7, Canada.

[7] Department of Medicine, Université de Montréal, Montreal, QC H3T 1J4, Canada.

*** Corresponding Author:** Anne-Marie Mes-Masson, Centre de recherche du Centre hospitalier de l'Université de Montréal, Montreal, QC H2X 0A9, Canada. Phone: 514-890-8000 ext. 25496; E-mail: anne-marie.mes-masson@umontreal.ca

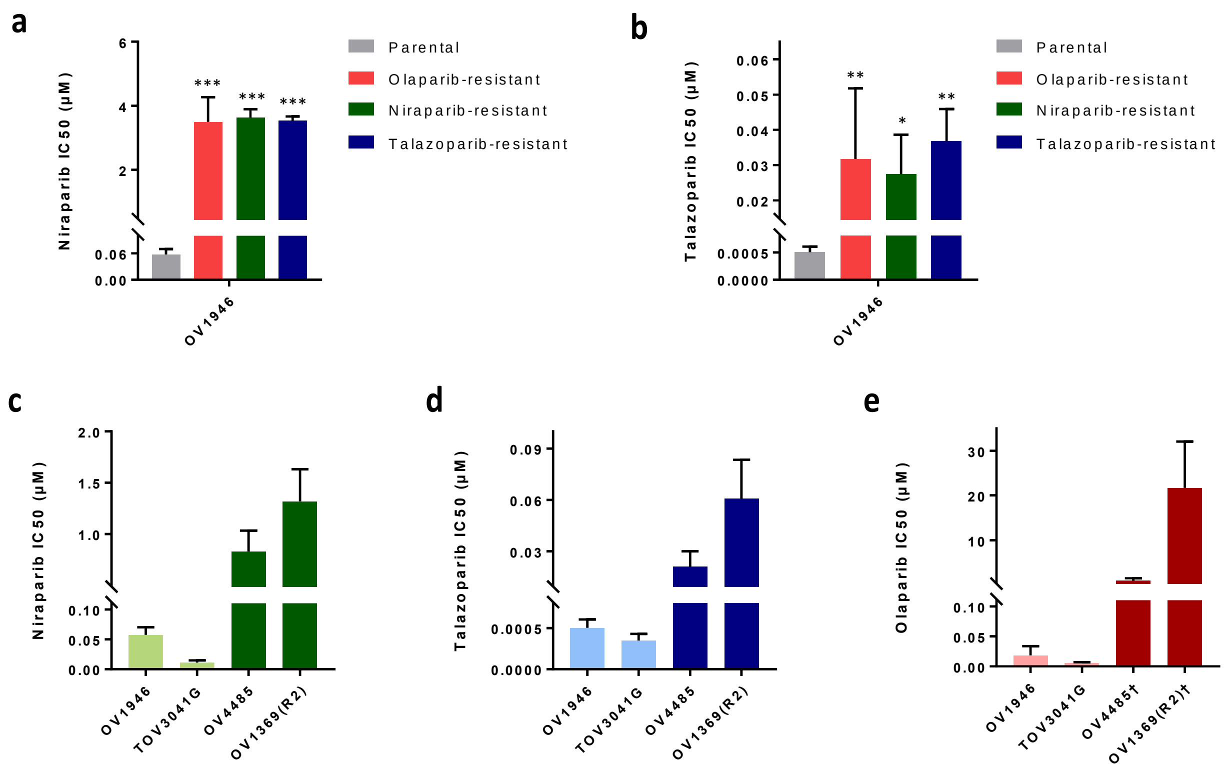

Supplementary Figure S1 – **Sensitivity of the resistant cell lines to niraparib and talazoparib.** Bar graphs of the OV1946 acquired resistance cell lines, and their parental counterpart, to niraparib (a) and talazoparib (b), as well as bar graphs illustrating the niraparib (c), talazoparib (d) and olaparib (e) sensitivity of OV4485 and OV1369(R2), in comparison to two sensitive cell lines. Experiments were repeated two to four times. Error bars represent SEM. Statistical significance was determined using Student's t-tests. *p<0.05, **p<0.01, ***p<0.001.

†Olaparib IC_{50} data for OV4485 and OV1369(R2) were taken from Fleury H, *et al.* (2017).

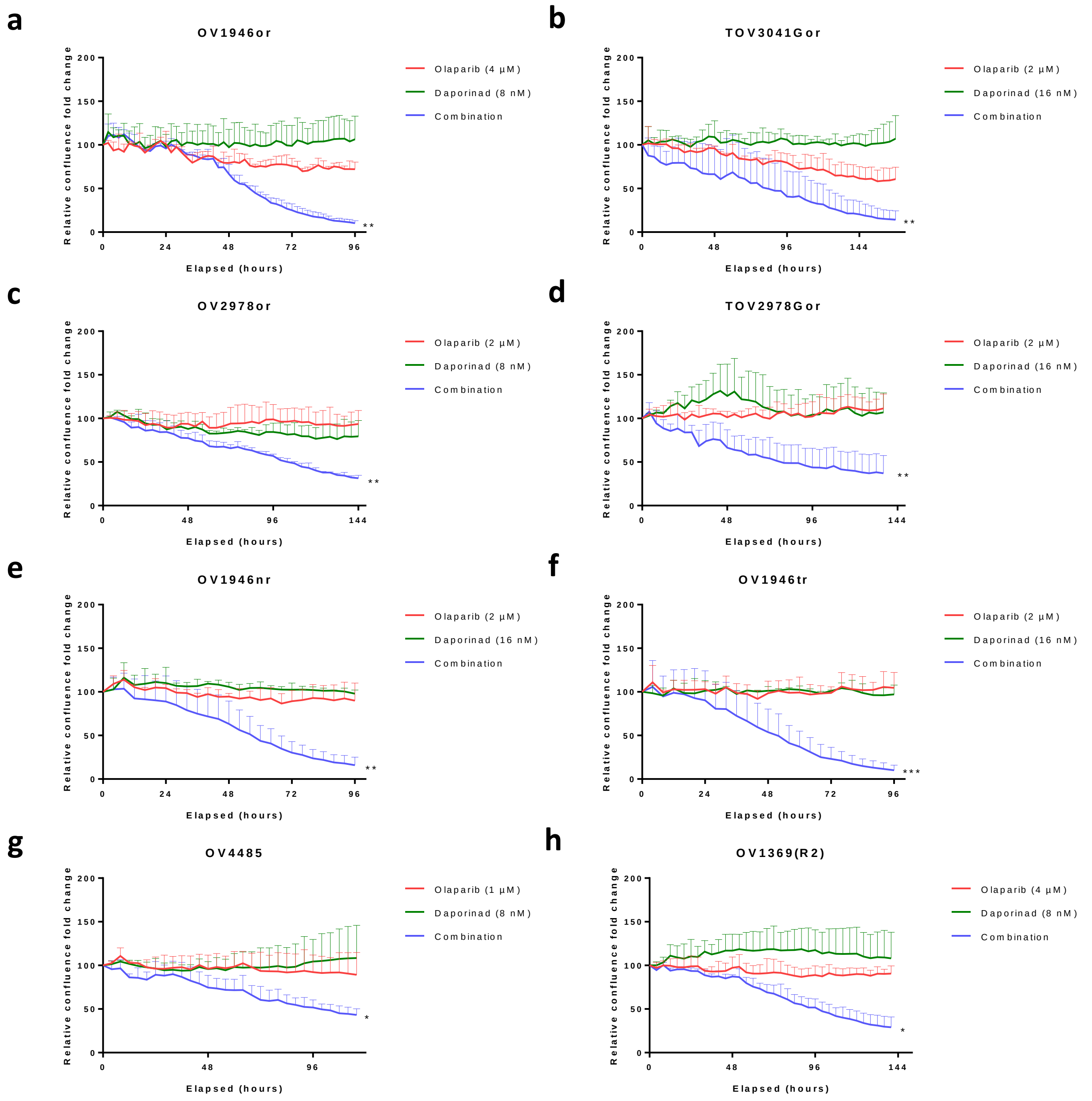

Supplementary Figure S2 – **Treatment growth curves of the combination of olaparib and daporinad.** Graphs representing confluence fold change over time per treatment, compared to vehicle (a-h). Experiments were repeated three times. Statistical significance was determined using Student's t-tests between the combination and each single agent, and only the highest p-value was illustrated. *p<0.05, **p<0.01, ***p<0.001. Error bars represent SEM.

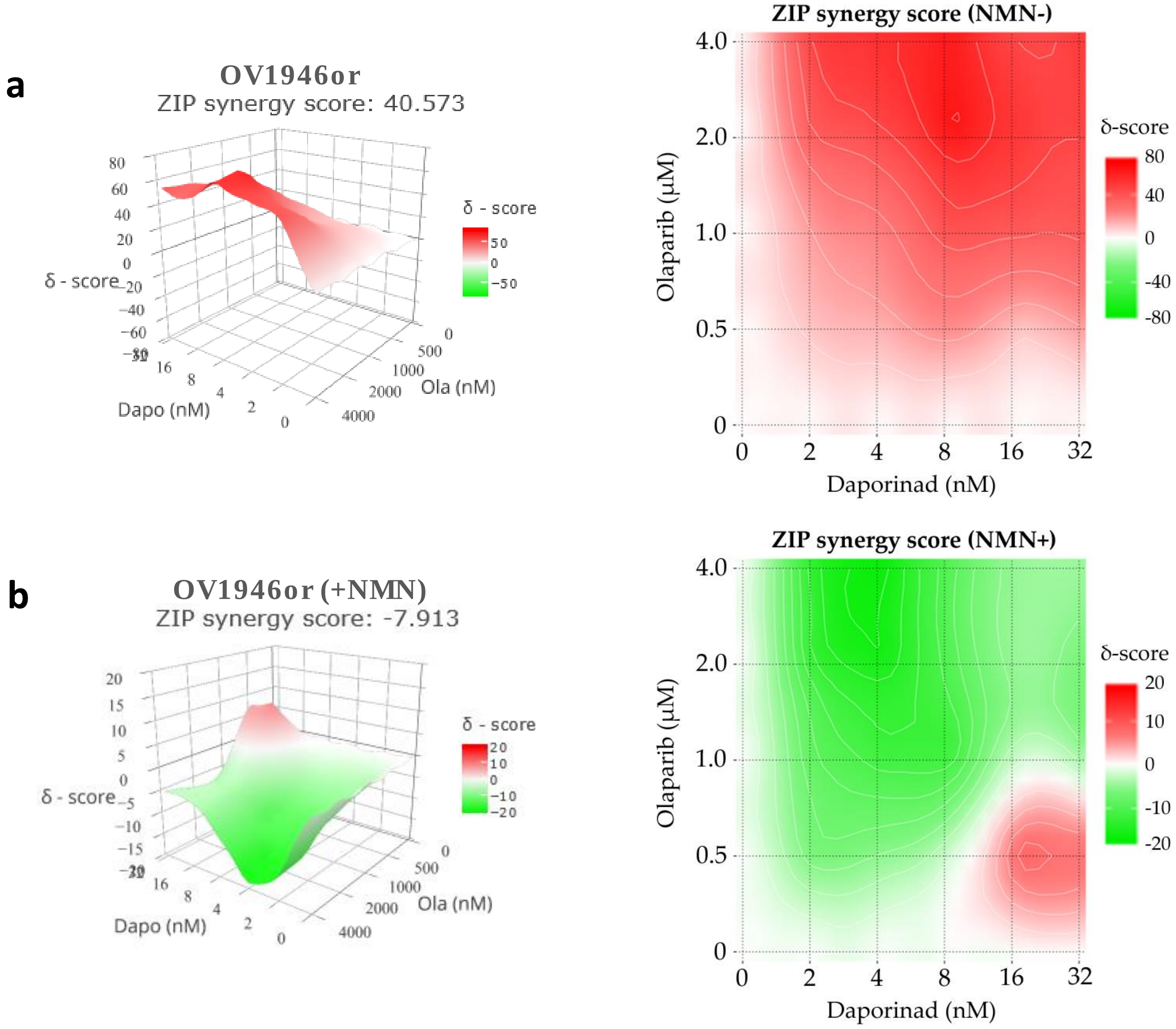

Supplementary Figure S3 – **Synergy map of the combination of olaparib and daporinad.** Representative graphical maps of the zero interaction potency (ZIP) scores of the combination of olaparib and daporinad in OV1946or. The same drug concentrations were used in absence (a) or presence (b) of NMN in the culture medium. Synergy is represented by red areas and positive zero interaction potency (ZIP) scores, while antagonism is represented by green areas and negative ZIP scores.

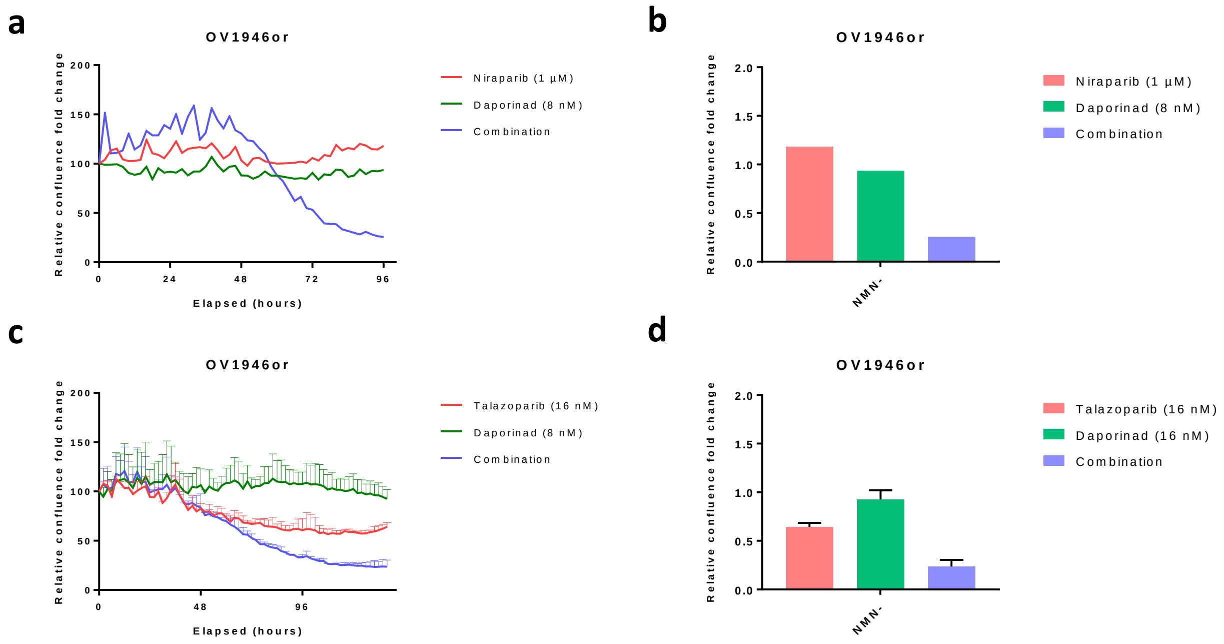

Supplementary Figure S4 – **Effect of the combination of daporinad and niraparib, and daporinad and talazoparib.** Live-cell imaging-based growth curves (a, c) and endpoints (b, d) of OV1946or treated with daporinad and niraparib (a, b), or daporinad and talazoparib (c, d). Experiments were done once (a, b) or twice (c, d). Error bars represent SEM.

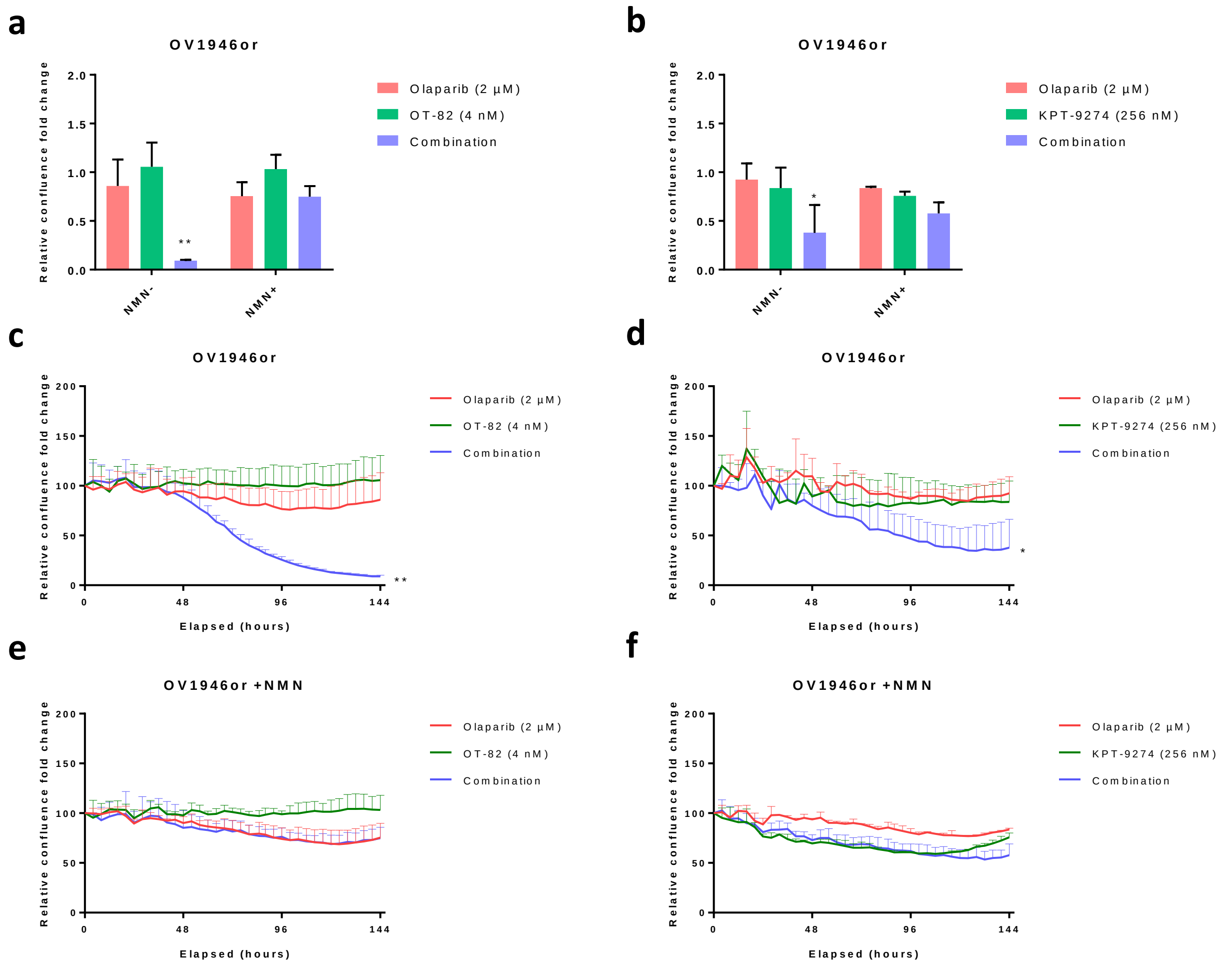

Supplementary Figure S5 – **Effect of treatment with olaparib and other NAMPT inhibitors.** Graphs representing the effect of treatment with olaparib in combination with NAMPT inhibitors OT-82 (a) and KPT-9274 (b). Confluence fold change over time per treatment, compared to vehicle, with (c-d) or without (e-f) NMN added into growth medium. Experiments were repeated two (f), three (d,e) or four (c) times. Statistical significance was determined using Student's t-tests between the combination and each single agent, and only the highest p-value was illustrated on graphs. *p<0.05, **p<0.01, ***p<0.001. Error bars represent SEM.

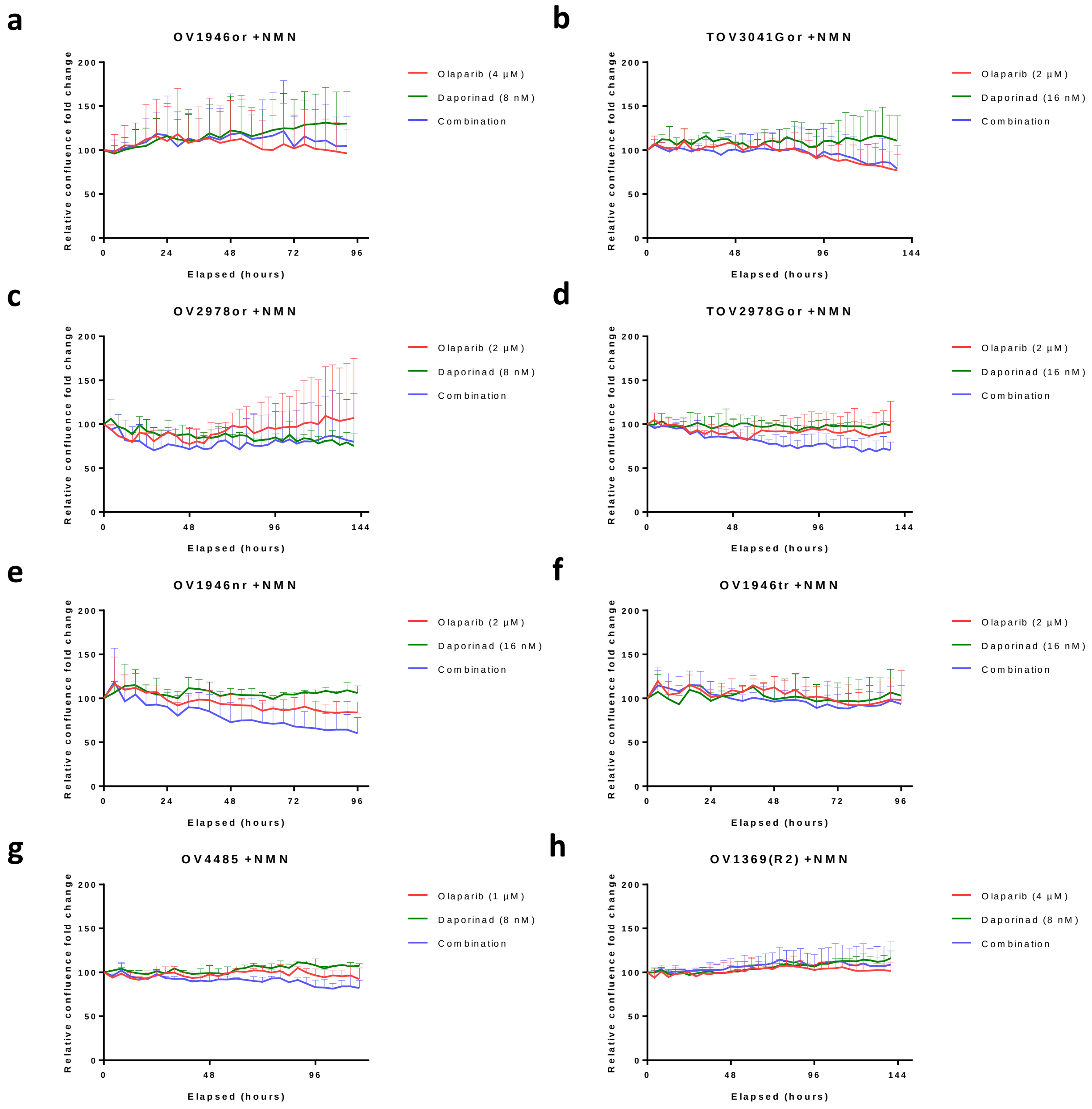

Supplementary Figure S6 – **Treatment growth curves of the combination of olaparib and daporinad, with added NMN.** Graphs representing confluence fold change over time per treatment, compared to vehicle, with NMN added into growth medium (a-h). Experiments were repeated three times. Statistical significance was determined using Student's t-tests between the combination and each single agent, and only the highest p-value was illustrated. Error bars represent SEM.

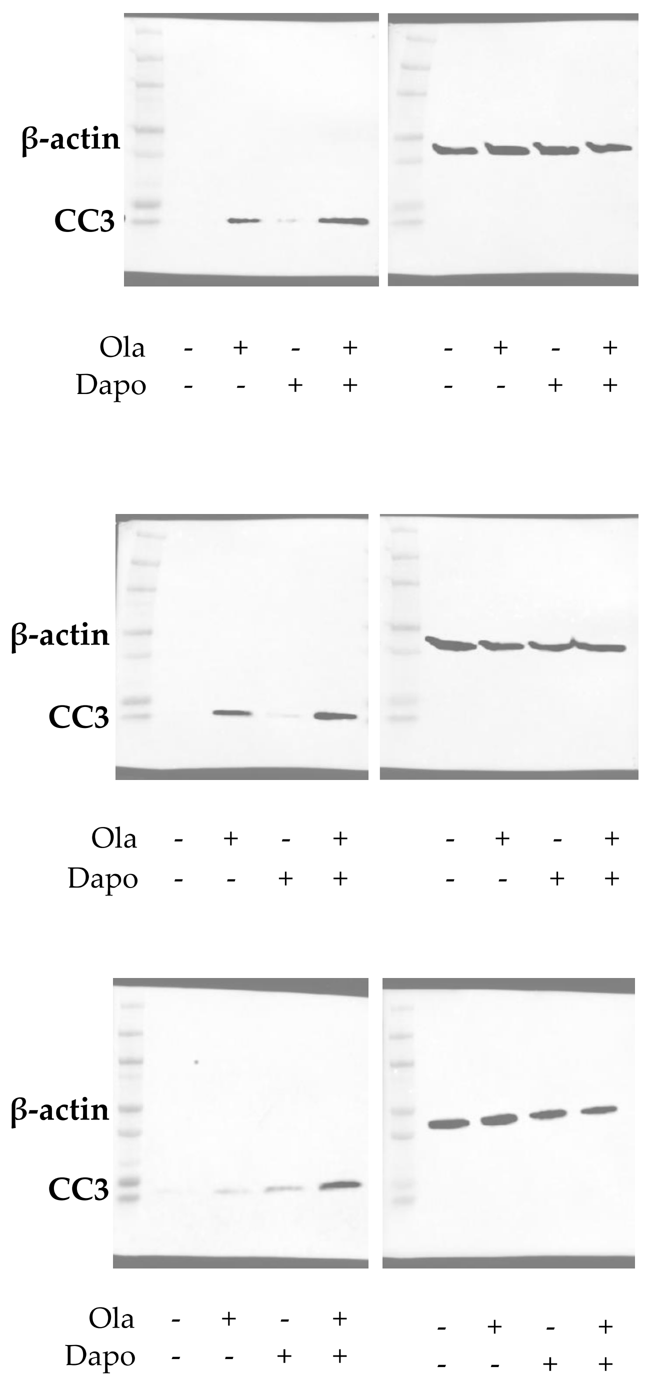

Supplementary Figure S7 – **Uncropped blots of Figure 3.** Blots are from three independent experiments that were used for CC3 quantification shown on Fig. 3c. Images on Fig. 3b are from the top set of blots. See methods for Western-Blot details.

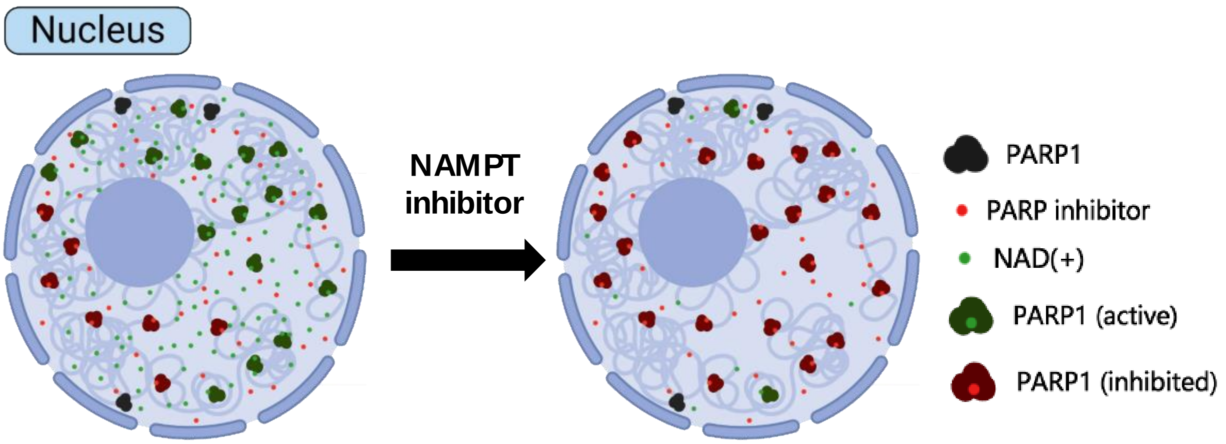

Supplementary Figure S8 – **Schematic model of NAMPT and PARP inhibitors combination therapy.** The NAMPT inhibitor induces NAD+ depletion in cancer cells. As a result, a greater proportion of PARP1 binds to the PARP inhibitor increasing DNA damage, inducing apoptosis and inhibiting tumor growth.

Supplementary Table S1. PARPi sensitivity of derived acquired resistance cell lines.

Parental cell line	Olaparib IC_{50} (µM)	Inhibitor used	Resistant cell line	Olaparib IC_{50} (µM)	Fold change
TOV3041G	0.006 ± 0.001 (n=3)	Niraparib	TOV3041Gnr	5.7 ± 2.2 (n=3)	950
		Talazoparib	TOV3041Gtr	4.8 ± 2.1 (n=3)	800
OV2978	0.006 ± 0.004 (n=3)	Niraparib	OV2978nr	2.4 (n=1)	400
		Talazoparib	OV2978tr	2.4 (n=1)	400
TOV2978G	0.01 ± 0.005 (n=3)	Niraparib	TOV2978Gnr	3.8 (n=1)	380
		Talazoparib	TOV2978Gtr	3.0 (n=1)	300
TOV1946	0.018 ± 0.015 (n=3)	Olaparib	TOV1946or	1.1 ± 0.2 (n=3)	61
		Niraparib	TOV1946nr	8.7 ± 2.8 (n=3)	485
OV2295	0.0013 (n=1)	Olaparib	OV2295or	1.5 ± 0.2 (n=2)	1154

Parental cell line	Niraparib IC_{50} (µM)	Inhibitor used	Resistant cell line	Niraparib IC_{50} (µM)	Fold change
OV1946	0.057 ± 0.013 (n=3)	Olaparib	OV1946or	3.5 ± 0.2 (n=3)	61.4
		Niraparib	OV1946nr	3.5 ± 0.8 (n=3)	61.4
		Talazoparib	OV1946tr	3.5 ± 0.1 (n=3)	61.4
TOV3041G	0.012 ± 0.004 (n=3)	Olaparib	TOV3041Gor	2.3 ± 0.7 (n=3)	191
		Niraparib	TOV3041Gnr	4.8 ± 3.2 (n=3)	400
		Talazoparib	TOV3041Gtr	3.9 ± 2.2 (n=3)	325

Parental cell line	Talazoparib IC_{50} (nM)	Inhibitor used	Resistant cell line	Talazoparib IC_{50} (nM)	Fold change
OV1946	0.463 ± 0.079	Olaparib	OV1946or	31.7 ± 11.2 (n=3)	68.4
		Niraparib	OV1946nr	27.4 ± 20.1 (n=3)	59.1
		Talazoparib	OV1946tr	34.8 ± 10.0 (n=3)	75.1
TOV3041G	0.035 ± 0.008 (n=3)	Olaparib	TOV3041Gor	39.0 ± 45.0 (n=2)	1114
		Niraparib	TOV3041Gnr	80.1 ± 71.4 (n=3)	2288
		Talazoparib	TOV3041Gtr	44.9 ± 34.4 (n=2)	1283

a. Experiments were repeated one to three times, as indicated in parentheses for each IC_{50} value.

Supplementary Table S2. Detailed endpoint fold changes of single drug versus combination without NMN.

Cell line	Olaparib (μM)	Daporinad (nM)	Olaparib versus combo		Daporinad versus combo	
			Fold change	*p-value*	*Fold change*	*p-value*
OV1946or	4	8	-7.0	0.00026	-10.4	0.00334
TOV3041Gor	2	16	-4.3	0.00927	-7.5	0.00484
OV2978or	2	8	-2.9	0.00215	-2.4	0.00926
TOV29878Gor	2	16	-3.0	0.00795	-2.9	0.01593
OV1946nr	2	16	-5.7	0.00450	-6.2	0.00014
OV1946tr	2	16	-10.5	0.00092	-9.7	0.00026
OV4485	1	8	-2.1	0.03934	-2.5	0.04160
OV1369(R2)	4	8	-3.1	0.00196	-3.7	0.01307

a. Experiments were repeated three times. Statistical significance was determined using Student's t-tests between the combination and each single agent.

Supplementary Table S3. Detailed endpoint fold changes of single drug versus combination for other NAMPT inhibitors.

Combination	Comparison	OV1946or		OV1946or +NMN 0.5 mM	
		Fold change	*p-value*	*Fold change*	*p-value*
Olaparib (2 μM) + OT-82 (4 nM)	Olaparib vs combo	-9.7	0.0013	-1.0	0.9681
	OT-82 vs combo	-11.9	0.0002	-1.4	0.0552
Olaparib (2 μM) + KPT-9274 (256 nM)	Olaparib vs combo	-2.4	0.0459	-1.4	0.0853
	KPT-9274 vs combo	-2.2	0.0098	-1.3	0.1711

a. Experiments were repeated three times. Statistical significance was determined using Student's t-tests between the combination and each single agent.

Supplementary Table S4. Detailed endpoint fold changes of single drug versus combination with NMN.

Cell line	Olaparib (μM)	Daporinad (nM)	Olaparib versus combo + (NMN 0.5 mM)		Daporinad versus combo + (NMN 0.5 mM)	
			Fold change	*p-value*	*Fold change*	*p-value*
OV1946or	4	8	1.1	0.747	-1.2	0.422
TOV3041Gor	2	16	1.5	0.641	1.0	0.976
OV2978or	2	8	-1.3	0.618	1.1	0.890
TOV29878Gor	2	16	-1.3	0.377	-1.4	0.010
OV1946nr	2	16	-1.4	0.131	-1.9	0.016
OV1946tr	2	16	-1.1	0.847	-1.1	0.648
OV4485	1	8	-1.1	0.329	-1.3	0.009
OV1369(R2)	4	8	1.1	0.681	-1.1	0.672

a. Experiments were repeated three times. Statistical significance was determined using Student's t-tests between the combination and each single agent.

Supplementary Table S5. Absolute mouse tumor volumes.

Group	Mouse ID	Tumor volume (mm^3)							
		Day 0	Day 2	Day 5	Day 8	Day 12	Day 15	Day 19	Day 22
Control	3812	434.7	460.08	564.376	657.272	777.92	871.2	1018.576	1267.2
Control	3819	560.64	564.48	697.2	707.919	861.738	1141.805	1169.792	1488.256
Control	3831	481.65	599.85	565.728	721.71	641.364	691.6	753.66	837.33
Control	3802	563.112	577.348	741.664	772.74	892.62	990.564	1215.136	1415.715
Control	3846	460.412	477.555	465.114	624.486	746.139	892.5	1194.375	1241.46
Control	3870	442.307	464.1	521.478	719.055	829.98	952.56	1005.33	1079.16
Control	3858	440.44	476.58	589.275	624.75	699.352	829.17	967.946	1008.99
Control	3860	492.936	560.272	672	781.2	916.432	1164.096	1450.899	1611.472
Olaparib	3811	474.336	612	453.492	619.353	674.96	740.784	902.46	1061.928
Olaparib	3800	345.72	370.11	371.628	593.56	506.94	824.694	877.8	994.84
Olaparib	3844	463.32	549.682	536.613	641.24	655.344	714.56	843.144	897.512
Olaparib	3859	392.768	418.88	466.416	744.48	647.838	782.46	894.88	1068.067
Olaparib	3834	507.13	516.596	546.7	838.86	734.16	880.88	1173.9	1175.04
Olaparib	3835	624.8	675.598	684.302	874.2	920.736	1333.48	1531.53	1636.099
Olaparib	3849	481.74	475.2	544.895	606.464	532.224	793.8	724.5	988.8
Olaparib	3850	452.64	555.37	581.581	620.16	786.6	789.36	969.76	1014.6
Daporinad	3820	340.256	573.44	611.226	627.912	690.3	673.92	975.8	997.56
Daporinad	3801	814.968	1013.232	906.87	1211.392	1598.688	1513.952	1656.2	2050.58
Daporinad	3825	443.004	569.244	716.184	909.696	945.952	1119.96	1367.62	1429.004
Daporinad	3845	484.946	567.72	573.804	613.184	655.2	922.32	1200.6	1625.888
Daporinad	3848	464.1	464.712	415.296	591.192	648	776.568	867.75	998.73
Daporinad	3867	489.6	549.185	579.04	671.46	649.632	669.024	721.344	774.457
Daporinad	3868	366.048	406.35	479.688	543.32	552.415	687.525	749.952	778.734
Daporinad	3871	511.68	637.884	571.242	829.92	657.324	795.564	821.712	935.874
Combo	3818	582.978	685.762	640.453	790.56	750.805	892.242	1063.53	1089.76
Combo	3821	366.624	319.2	450.24	428.904	454.3	650.26	650.26	747.684
Combo	3836	467.84	534.198	603.288	746.76	867.744	765	1029.588	1099.56
Combo	3824	472.752	469.755	441.6	442.368	466.2	525.798	622.71	486.328
Combo	3832	342.55	349.44	384.54	402.402	320.044	386.745	455.868	463.32
Combo	3833	464.64	559.02	564.096	536.796	542.412	544.5	461.094	564.408
Combo	3869	479.88	483.003	536.18	494.856	485.76	509.184	458.325	469.092
Combo	3847	364.32	473.824	495.43	565.212	646.425	753.984	652.848	746.13
